# TongueNet: a multi-modal fusion and multi-label classification model for traditional Chinese Medicine tongue diagnosis

**DOI:** 10.3389/fphys.2025.1527751

**Published:** 2025-04-25

**Authors:** Lijuan Yang, Qiumei Dong, Da Lin, Xinliang Lü

**Affiliations:** ^1^ Department of Rheumatology, Inner Mongolia Autonomous Region Hospital of Traditional Chinese Medicine, Hohhot, China; ^2^ College of Traditional Chinese Medicine, Inner Mongolia Medical University, Hohhot, China; ^3^ School of Mathematical Sciences, Inner Mongolia University, Hohhot, China

**Keywords:** traditional Chinese Medicine (TCM), multimodal fusion, tongue diagnosis, deep learning, multi-label classification

## Abstract

Tongue diagnosis in Traditional Chinese Medicine (TCM) plays a crucial role in clinical practice. By observing the shape, color, and coating of the tongue, practitioners can assist in determining the nature and location of a disease. However, the field of tongue diagnosis currently faces challenges such as data scarcity and a lack of efficient multimodal diagnostic models, making it difficult to fully align with TCM theories and clinical needs. Additionally, existing methods generally lack multi-label classification capabilities, making it challenging to simultaneously meet the multidimensional requirements of TCM diagnosis for disease nature and location. To address these issues, this paper proposes TongueNet, a multimodal deep learning model that integrates tongue image data with text-based features. The model utilizes a Hierarchical Aggregation Network (HAN) and a Feature Space Projection Module to efficiently extract and fuse features while introducing consistency and complementarity constraints to optimize multimodal information fusion. Furthermore, the model incorporates a multi-scale attention mechanism (EMA) to enhance the diversity and accuracy of feature weighting and employs a Kolmogorov-Arnold Network (KAN) instead of traditional MLPs for output optimization, thereby improving the representation of complex features. For model training, this study integrates three publicly available tongue image datasets from the Roboflow platform and enlists multiple experts for multimodal annotation, incorporating multi-label information on disease nature and location to align with TCM clinical needs. Experimental results demonstrate that TongueNet outperforms existing models in both disease nature and disease location classification tasks. Specifically, in the disease nature classification task, it achieves 89.12% accuracy and an AUC of 83%; in the disease location classification task, it achieves 86.47% accuracy and an AUC of 81%. Moreover, TongueNet contains only 32.1 M parameters, significantly reducing computational resource requirements while maintaining high diagnostic performance. TongueNet provides a new approach for the intelligent development of TCM tongue diagnosis.

## 1 Introduction

As a comprehensive medical theory and practical system, Traditional Chinese Medicine (TCM) has a long history and holds an important place in Eastern medicine (Chiu, 2000; Chengdong et al., 2022). Its unique theories of syndrome differentiation and treatment, holistic approach, and diverse diagnostic methods form a systematic framework for diagnosing and treating diseases. Tongue diagnosis, one of the “Four Diagnoses” in TCM, involves observing the patient’s tongue characteristics—such as shape, color, and coating—to assess health status. Different tongue appearances reflect, to some extent, the state of internal organ function and blood circulation, providing unique support for clinical diagnosis and treatment (Li et al., 2022a; Li et al., 2022b; Qu et al., 2017).

In recent years, with breakthroughs in deep learning for image recognition, artificial intelligence has gradually been applied in tongue diagnosis (Zhang et al., 2022; Tang et al., 2020). Through image recognition and deep learning models, tongue images can be analyzed automatically, advancing the digitization and standardization of tongue diagnosis. These studies use deep neural networks to analyze tongue image data, offering objective, quantifiable support that enhances diagnostic accuracy (Fan et al., 2021). However, most research is limited to single-modality analysis of tongue images, which fails to capture the complexity and complementary nature of the multi-source information integral to TCM diagnosis (Xu et al., 2020). Specifically, single-modality analysis primarily relies on extracting features from tongue images, which can reflect pathological states to a certain extent but overlooks complementary diagnostic information, such as the textual description of the tongue diagnosis. Data from a single modality often cannot fully represent the patient’s complex health condition, which limits diagnostic model performance (Jiang et al., 2021). Additionally, due to visual similarities among tongue features, image-only analysis may fail to distinguish subtle pathological differences, affecting diagnostic accuracy and precision (Fan et al., 2021).

Current AI-based tongue diagnosis research faces three major challenges: First, there is a severe lack of data for Traditional Chinese Medicine (TCM) tongue diagnosis, particularly the absence of paired multi-modal data, which limits the effective development of multi-dimensional tongue diagnosis analysis (Wang et al., 2020). Second, most existing AI diagnostic models are based on general machine learning algorithms or Western medical imaging frameworks, failing to fully integrate the unique information structure and diagnostic logic of TCM tongue diagnosis. This results in insufficient accuracy and interpretability when processing TCM diagnostic data (Balasubramaniyan et al., 2022). Lastly, there is a lack of multi-label classification models specifically for TCM tongue diagnosis, which restricts the ability to perform comprehensive, multi-dimensional, and multi-perspective analysis and classification of tongue features (Jiang et al., 2021).

To address the above issues, this paper proposes a new multimodal deep learning model—TongueNet. First, from a data construction perspective, a high-quality, multimodal TCM tongue diagnosis dataset is systematically established, encompassing tongue image data and corresponding textual annotations, along with multi-label classification tags for disease type and disease location. This ensures the multidimensionality and comprehensiveness of the dataset. Next, the TongueNet model fuses tongue image data with textual information by leveraging multimodal learning techniques to deeply integrate image and text features. Through the introduction of innovative methods such as consistency and complementarity constraints, the model effectively combines the advantages of both modalities, avoiding potential information loss or bias that might arise from using a single modality. Furthermore, TongueNet adopts a multi-label classification framework, enabling the simultaneous recognition and classification of multiple related tongue features, thus supporting a multidimensional and multi-perspective analysis and diagnosis of tongue images.

The contributions of this paper are as follows:

• This paper integrates three tongue diagnosis datasets from the Roboflow platform to construct a high-quality multimodal dataset containing tongue image data and corresponding text annotations, with multi-label classification tags for pathology and disease location. This dataset not only addresses the shortage of multimodal data in the field of TCM tongue diagnosis but also provides rich, multidimensional data support for subsequent tongue feature analysis and model training.

• This paper proposes TongueNet, an innovative multimodal deep learning model that simultaneously integrates features from tongue image data and text descriptions. By employing a HAN and feature space projection modules, TongueNet efficiently extracts and fuses multimodal information, thereby enhancing diagnostic capability and clinical applicability in tongue diagnosis.

• This paper creatively incorporates a EMA, allowing TongueNet to focus more precisely on key pathological features in tongue images and optimize the handling of information at different scales. This improvement significantly enhances the model’s diagnostic accuracy, especially in processing complex tongue diagnosis images.

• This paper replaces the traditional MLP model with the KAN, enabling TongueNet to more effectively represent and process complex tongue diagnosis features. Experimental results show that this enhanced feature representation capability allows TongueNet to achieve higher accuracy in tongue image classification tasks.

• Experimental results demonstrate that TongueNet significantly outperforms existing traditional models in tongue diagnosis tasks.

## 2 Related work

### 2.1 Multimodal fusion strategy

In recent years, multimodal fusion has been extensively studied in fields such as medical imaging and emotion recognition (Azam et al., 2022). Multimodal fusion methods generally include early fusion, intermediate fusion, and late fusion. Early fusion combines the raw features of different modalities directly at the data input stage, effectively leveraging the initial associations between modalities to form a unified representation space (Hermessi et al., 2021). In medical imaging, some studies have combined image and text data after feature extraction for diagnosis, fully utilizing the spatial information from images and semantic information from text to enhance diagnostic accuracy (Tan et al., 2020). Other studies have fused various physiological data at the feature level to analyze complex conditions, achieving multi-dimensional diagnosis. However, these methods have limited capacity for deep modality relationship exploration, are prone to introducing data noise, and often fall short in comprehensive feature analysis (Huang B. et al., 2020). Intermediate fusion integrates different modality features at intermediate layers after preliminary extraction to capture complementary information between modalities more fully (Tang W. et al., 2022; Tawfik et al., 2021). Certain disease prediction models have achieved a significantly higher cross-modal utilization rate by fusing image and text data from patients within hidden layers. Additionally, in emotion analysis, integrating image and audio features at intermediate layers has led to high accuracy. Although intermediate fusion can better explore deep relationships between modalities, it is computationally complex and requires substantial computing resources (Alseelawi et al., 2022; Singh et al., 2024). Late fusion combines decisions after each modality has been processed independently, making it suitable for tasks that demand high stability and robustness. For instance, multimodal tumor recognition methods independently process CT images and pathology reports before weighted fusion, ensuring diagnostic robustness. In emotion recognition, late fusion of voice and video features enhances recognition accuracy, demonstrating good adaptability. However, this method cannot fully exploit deep intermodal relationships, making its fusion of information less comprehensive than early or intermediate fusion (Li et al., 2021; Yadav and Yadav, 2020). In TCM tongue diagnosis, the complex associations between different modalities are difficult to uncover using traditional methods.

This paper proposes a novel multimodal fusion model, TongueNet, which constructs a multimodal diagnostic framework aligned with TCM theory by combining tongue image and text information and applying consistency and complementarity constraints in the representation space. This approach improves diagnostic accuracy and clinical applicability.

### 2.2 Intelligent medical auxiliary diagnosis

In recent years, with the rapid development of artificial intelligence technologies, advanced techniques such as deep learning have seen increasing applications in the medical field, particularly in intelligent medical auxiliary diagnosis, where significant progress has been made (Wang et al., 2021). In the field of medical imaging, deep learning models, especially Convolutional Neural Networks (CNN) and Residual Networks (ResNet), have been widely applied for auxiliary diagnosis (Xu et al., 2020). For example, CNNs are used to extract high-dimensional features from medical images, significantly improving the accuracy of image analysis (Xu H. et al., 2021). In tasks like image segmentation and tumor recognition, CNNs have demonstrated outstanding performance, achieving notable results in fields such as lung CT scans and breast cancer screening. ResNet, on the other hand, addresses the vanishing gradient problem in deep networks by introducing residual connections, improving model performance and stability (Lin et al., 2020; Xu H. et al., 2021). Additionally, Transfer Learning, as an efficient learning method, has also been widely used in medical imaging, particularly for situations with insufficient data or challenging annotations, by transferring knowledge from existing models to enhance the generalization ability of the model (Xu Q. et al., 2021). Beyond medical imaging, intelligent medical diagnosis has started to expand into the domain of sound signals. Multi-class classification of sound signals, using traditional machine learning methods such as Support Vector Machines (SVM) and K-Nearest Neighbors (KNN), has also been applied for auxiliary diagnosis (Naeem et al., 2021). For example, by analyzing features such as a patient’s voice signals and respiratory sounds, intelligent diagnostic systems can effectively assist doctors in initial disease screening and diagnosis (Garg and Mago, 2021). In the diagnosis of certain neurological disorders, voice analysis has shown great potential, particularly in early diagnosis of cognitive impairments, Alzheimer’s disease, and other conditions, where changes in voice signals can serve as an important diagnostic clue (Gupta et al., 2021).

However, despite the achievements of single-modality methods in disease diagnosis, multimodal fusion remains crucial in intelligent healthcare applications. In this paper, we utilize representation learning to learn shared representations of different modalities within the data, effectively integrating information from different sources to enhance the diagnostic capability of the model.

### 2.3 Intelligent tongue diagnosis

Intelligent tongue diagnosis, as an important component of TCM diagnosis, combines tongue and facial feature information and integrates with TCM theory to offer new approaches and methods for disease diagnosis (Chengdong et al., 2022). Tongue diagnosis involves observing features such as the shape, color, and coating of the tongue, which can reflect the health status of the body. In recent years, with the development of computer vision and artificial intelligence technologies, intelligent tongue diagnosis has gradually become a convenient and accurate auxiliary diagnostic tool (Mukai et al., 2022). In the early stages of tongue diagnosis research, traditional machine learning methods, such as SVM and KNN, were widely used for classifying tongue texture and coating features (Balu and Jeyakumar, 2021). These methods extracted features such as texture, color, and shape from tongue images and combined them with patient information such as age, gender, and medical history for disease diagnosis. For example, SVM-based tongue coating texture analysis methods can extract texture features from the tongue coating to analyze and determine the disease type, offering a more accurate diagnosis when combined with traditional TCM theory (Song et al., 2020). KNN algorithms have also been used to analyze features such as the shape and color of the tongue to help classify different conditions. However, these methods still face certain challenges when processing complex tongue diagnosis images, such as feature extraction accuracy and image complexity (Song et al., 2020; Li J. et al., 2022). With the development of deep learning techniques, CNN have gradually become mainstream in the field of tongue diagnosis (Yuan and Liao, 2020). CNNs automatically extract deep features from tongue diagnosis images, offering significant advantages over traditional methods. Since tongue and facial images are mostly visible light images, CNNs can effectively capture subtle changes in the images, enabling accurate analysis of TCM constitution and diseases. For instance, CNN-based automated analysis of tongue texture, color, and shape has greatly improved the efficiency and accuracy of tongue diagnosis, especially when trained on large-scale datasets, where CNNs show strong generalization ability. In recent years, Transformer models have also been applied in the field of tongue diagnosis, particularly in the analysis of tongue and facial feature points. Transformers can effectively capture dependencies between different regions and use self-attention mechanisms to weight information from different parts of the tongue diagnosis images, further improving diagnostic accuracy (Huang X. et al., 2020). By jointly analyzing tongue and facial feature points, Transformers enable more comprehensive health assessments, providing new technical support for intelligent tongue diagnosis systems.

## 3 Methodology

### 3.1 Overview

**FIGURE 1:**
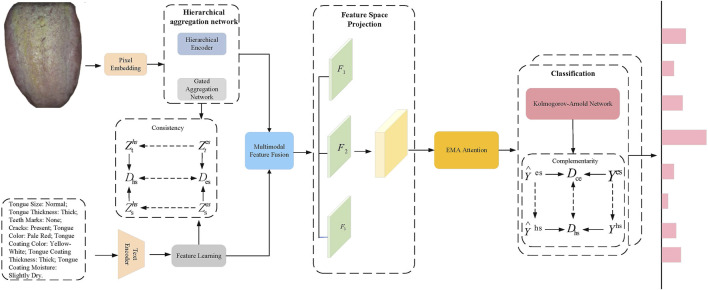
TongueNet Overall Network Architecture Diagram. The image features are extracted using a hierarchical aggregation network, while the text features are obtained through a text encoder. Modal fusion is performed via consistency constraints, followed by a feature space projection module where the fused features are further weighted using the EMA attention mechanism. Finally, classification prediction is made through the KAN network.

In this paper, we propose the TongueNet network, a framework designed to fully leverage the consistency and complementarity between multimodal data. Through multi-level feature extraction, cross-modal fusion, spatial mapping and projection, and attention mechanisms, the network aims to enhance the diagnostic model’s accuracy and generalization ability. As shown in Figure 1, we first construct independent feature extraction networks for unimodal data (tongue images and text). Tongue image data is processed through a hierarchical aggregation network (comprising a hierarchical encoder and a global aggregation network) to extract modality-specific features, while text data is fed into a neural transformation network to extract personalized features specific to the text modality. These features are initially represented in Euclidean space to capture each modality’s unique characteristics. To achieve spatial alignment and feature fusion across modalities, we designed a feature space transformation module, which maps unimodal features from Euclidean space to hyperbolic space, where cross-modal consistency is calculated and optimized. Next, a cross-modal bridging strategy is employed to fuse tongue image and text features, capturing and reinforcing the complementarity between multimodal features. The fused features are then fed into a feature space projection module, where they are projected into three independent yet complementary subspaces to generate feature representations with complementary information. During this process, an Multi-scale attention (EMA) Ouyang et al. (2023) attention mechanism is applied to further weight the fused features, emphasizing the synergy between different modalities and enhancing the diversity and effectiveness of the feature representations. Finally, the fused multimodal features are input into the stacked KAN layers to obtain label predictions in Euclidean space. To further optimize classification accuracy, the label predictions are mapped to hyperbolic space. By calculating the loss between the predicted values and the true labels in both Euclidean and hyperbolic spaces, dual optimization for multi-label prediction is achieved. This process leverages the complementarity of Euclidean and hyperbolic spaces in feature representation, thereby enhancing the model’s accuracy and robustness in complex diagnostic tasks, meeting the clinical needs of TCM diagnosis.

### 3.2 Hierarchical aggregation network

**FIGURE 2:**
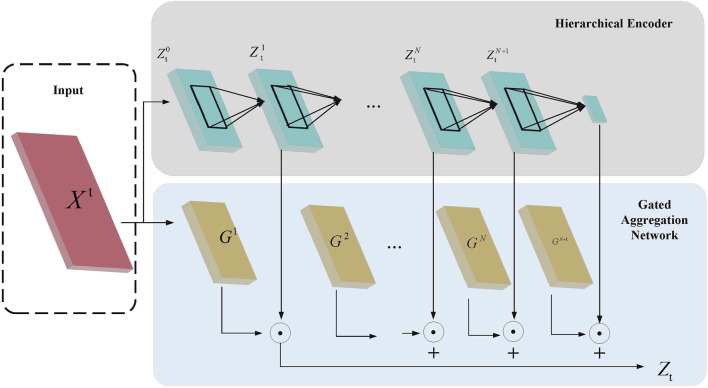
Hierarchical Aggregation Network Architecture Diagram. The diagram illustrates the process where the input features $X^{\prime }$ are first processed through the Hierarchical Encoder to generate feature representations $Z^{\left(0\right)},Z^{\left(1\right)},\ldots ,Z^{\left(n\right)}$ . These features are then aggregated using the Gated Aggregation Network, which combines them into the final feature representation $Z_{l}$ .

For processing tongue image features, we propose the Hierarchical Aggregation Network (HAN), which, as shown in Figure 2, includes two key modules: the hierarchical encoder module and the gated aggregation network module.

In the Hierarchical Encoder module, assume the input feature is $X_{t}$ . First, a linear layer $f_{0}$ is applied to map the input feature into the hidden layer space, resulting in

$$Z_{t}^{0}=f_{0}\left(X_{t}\right)$$

Then, a group convolution layer with N groups is used to extract features at different hierarchical levels, obtaining the hierarchical feature representations $Z_{t}^{N}$ , as follows:

$$Z_{t}^{N}=f_{N}\left(Z_{t}^{N-1}\right),\;\left(N\in \left[1,N\right]\right)$$

where $f_{N}$ represents a group convolution operation. Group convolution reduces the number of parameters while maintaining channel independence, which is beneficial for capturing broader contextual information. For example, when the convolution kernel size is k(N) and the stride is s(i) , the receptive field at different levels expands with the hierarchy. The receptive field size at level N is given by:

$$l\left(N\right)=l\left(N-1\right)+\left[\left(k\left(N\right)-1\right)\times \overset{N-1}{\underset{i=1}{\prod }}s\left(i\right)\right]$$

To enhance global contextual information, a global average pooling operation is applied at the final level of the hierarchical encoder, yielding the final hierarchical feature $Z_{t}^{N+1}$ :

$$Z_{t}^{N+1}=f_{\mathrm{gap}}\left(Z_{t}^{N}\right)$$

The Gated Aggregation Network module assigns weights to features of different granularities, denoted by $G=\left\{G^{1},G^{2},\ldots ,G^{N},\ldots ,G^{N+1}\right\}$ , which represents the importance of each layer’s features. The weights are calculated using a function $f_{G}\left(X\right)$ , with an output dimension of $R^{H\times W\times \left(N+1\right)}$ . Finally, the aggregation of features at different granularities is achieved by performing element-wise multiplication with each hierarchical feature and summing the results, computed as:

$$Z_{t}=\overset{N+1}{\underset{N=1}{\sum }}G^{N}⊙Z_{t}^{N}$$

where ⊙ denotes element-wise multiplication, defined as:

$$G^{N}⊙Z_{t}^{N}=\left[\begin{array}{ccc} {\left[G^{N}\right]}_{0,0}\cdot {\left[Z_{t}^{N}\right]}_{0,0} & \cdots & {\left[G^{N}\right]}_{0,j}\cdot {\left[Z_{t}^{N}\right]}_{0,j}\\ \vdots & \ddots & \vdots \\ {\left[G^{N}\right]}_{i,0}\cdot {\left[Z_{t}^{N}\right]}_{i,0} & \cdots & {\left[G^{N}\right]}_{i,j}\cdot {\left[Z_{t}^{N}\right]}_{i,j} \end{array}\right]$$

Through this hierarchical encoding and gated aggregation strategy, HAN can extract feature information at different scales and adaptively aggregate information by assigning different weights, thereby effectively enhancing the model’s representational capacity in multi-modal feature fusion.

### 3.3 Feature space projection

In the feature space projection module, the fused multimodal feature F is projected onto multiple complementary subspaces to enhance the feature representation capability. Specifically, the fused feature F is mapped to three different subspaces $S_{1}$ , $S_{2}$ , and $S_{3}$ , generating three feature representations $F_{1}$ , $F_{2}$ , and $F_{3}$ with complementary information. The equations are as follows:

$$\left\{\begin{array}{c} F_{1}=W_{1}F+b_{1}\;\\ F_{2}=W_{2}F+b_{2}\;\\ F_{3}=W_{3}F+b_{3}\; \end{array}\right.$$

where $W_{1}$ , $W_{2}$ , and $W_{3}$ are the projection matrices, and $b_{1}$ , $b_{2}$ , and $b_{3}$ are the corresponding bias terms. Each subspace is designed with unique projection matrices and biases to capture complementary information from multiple dimensions of the multimodal features. Through this multi-dimensional feature decomposition, the feature space projection module enhances the diversity and complementarity of the feature representations within the model.

### 3.4 Kolmogorov-arnold network

In this paper, we employ the Kolmogorov-Arnold Network (KAN) in place of the traditional MLP as the final classifier. The primary motivation for this replacement lies in KAN’s superior expressive power and interpretability. Compared to MLP, KAN is based on the Kolmogorov-Arnold representation theorem, where learnable activation functions are placed on the edges instead of fixed node activations, enabling the network to capture complex relationships in data more flexibly and efficiently.

Specifically, KAN does not use traditional linear weight matrices but replaces each weight parameter with a univariate function. This transforms the “linear transformation + nonlinear activation” structure in MLP into a “direct combination of nonlinear activations,” simplifying the computation and enhancing parameter efficiency. The KAN designed in this work consists of multiple layers of functions, where the activation value $x_{l+1,j}$ at each layer is computed from the input $x_{l,i}$ of the previous layer using the activation function $\varphi_{l,j,i}$ . The equations are as follows:

The calculation of activation values at each layer:

$$x_{l+1,j}=\overset{n_{l}}{\underset{i=1}{\sum }}\varphi_{l,j,i}\left(x_{l,i}\right)$$

The process of combining multiple layers to form the final output:

$$KAN\left(x\right)=\left(\Phi_{L-1}◦\Phi_{L-2}◦\cdots ◦\Phi_{1}◦\Phi_{0}\right)\left(x\right)$$

The final form of the output equation:

$$f\left(x\right)=\overset{n_{L-1}}{\underset{i_{L-1}=1}{\sum }}\varphi_{L-1,i_{L-1}}\left(\overset{n_{L-2}}{\underset{i_{L-2}=1}{\sum }}\cdots \overset{n_{1}}{\underset{i_{1}=1}{\sum }}\varphi_{1,i_{2},i_{1}}\left(\overset{n_{0}}{\underset{i_{0}=1}{\sum }}\varphi_{0,i_{1},i_{0}}\left(x_{i_{0}}\right)\right)\cdots \;\right)$$

By using KAN instead of MLP for the final classification, we leverage KAN’s capability in representing high-dimensional features, while its finer-grained activation functions allow the learning of more complex feature patterns. Moreover, KAN’s layer-by-layer structure enhances interpretability, which aligns well with the need for feature expressiveness in complex diagnostic tasks. This structure not only improves model accuracy but also meets the requirements of multimodal feature representation in TCM diagnosis.

### 3.5 Multi-scale attention

**FIGURE 3:**
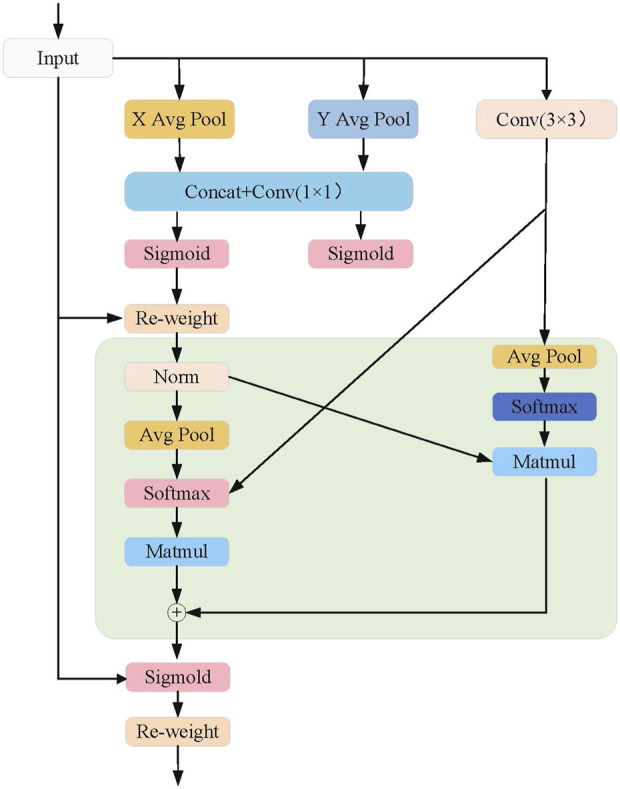
EMA module network architecture diagram.

The design of the EMA attention mechanism aims to enhance feature representation capability while reducing computational complexity. This mechanism divides the channel dimension into multiple sub-feature groups, allowing spatial semantic information to be evenly distributed across each group. As shown in Figure 3, the core steps of the EMA attention mechanism are as follows: 1) Channel Division and Sub-feature Group Construction: The EMA attention mechanism first divides the channel dimension into multiple sub-feature groups. This division enables each sub-feature group to capture its internal spatial information distribution with lower computational cost. Additionally, this approach allows for more fine-grained feature processing, enabling each sub-feature group to effectively retain spatial semantic information. 2) Global Information Encoding: Before further processing the feature groups, the EMA attention mechanism performs global information encoding on the input features. Global information, obtained through global average pooling, is used to recalibrate the channel weights within each feature group. This allows the model to focus more on the feature regions contributing to the final result, while ignoring irrelevant or noisy features. 3) Cross-dimensional Interaction: The EMA mechanism further aggregates the output features of two parallel branches through cross-dimensional interaction. Specifically, two parallel branches process different sub-feature groups and then fuse information across dimensions. This cross-dimensional interaction design enhances the complementarity between features, enabling the fused features to better represent the input multimodal information.

Specifically, we first perform aggregation along the height dimension, as shown in the formula below:

$$z_{c}^{H}\left(H\right)=\frac{1}{W}\underset{0\leq i\leq W}{\sum }x_{c}\left(H,i\right)$$

where $z_{c}^{H}\left(H\right)$ represents the mean feature aggregation of channel c along the height dimension H , and $x_{c}\left(H,i\right)$ is the feature value of channel c at height H and width i . W is the width of the feature map. This step averages over the width dimension to obtain global information for each channel along the height dimension.

Next, we perform aggregation along the width dimension:

$$z_{c}^{W}\left(W\right)=\frac{1}{H}\underset{0\leq j\leq H}{\sum }x_{c}\left(j,W\right)$$

where $z_{c}^{W}\left(W\right)$ represents the mean feature aggregation of channel c along the width dimension W , and $x_{c}\left(j,W\right)$ is the feature value of channel c at height j and width W . H is the height of the feature map. This step averages over the height dimension to obtain the global feature information of each channel along the width dimension.

Next, we perform global spatial aggregation by averaging the feature values at all spatial positions:

$$z_{c}=\frac{1}{H\times W}\overset{H}{\underset{j=0}{\sum }}\overset{W}{\underset{i=0}{\sum }}x_{c}\left(i,j\right)$$

where $z_{c}$ is the global average feature value of channel c across the entire spatial space, representing the global information of this channel over the entire feature map.

The cross-dimensional interaction strategy of the EMA attention mechanism further enhances the robustness and generalization ability of the diagnostic model in TongueNet. By interacting the outputs of parallel branches, the EMA mechanism effectively reduces redundant information, allowing the model to maintain high diagnostic accuracy while distinguishing subtle pathological features more precisely within the multimodal feature space.

### 3.6 Loss function

In this method, to enhance the consistency and complementarity of multimodal features across different representation spaces, consistency and complementarity loss functions are designed to optimize the feature space, ultimately improving the model’s diagnostic accuracy and robustness.

Firstly, the consistency of feature representation space ensures that similar feature vectors remain consistent across different representation spaces, meaning that distance metrics in different spaces should be similar. Specifically, let the distinctive feature of the tongue image, after being processed by the hierarchical aggregation network, be represented in Euclidean space as $Z_{t}^{es}$ and in hyperbolic space as $Z_{t}^{hs}$ . Likewise, the text features, after being processed by the neural transformation network, are represented in Euclidean space as $Z_{s}^{es}$ and in hyperbolic space as $Z_{s}^{hs}$ . To achieve spatial consistency, a consistency loss function $L_{\text{consis}}$ is defined as follows:

$$L_{\text{consis}}=1-\frac{D_{es}\left(Z_{t}^{es},Z_{s}^{es}\right)\cdot D_{hs}^{c}\left(Z_{t}^{hs},Z_{s}^{hs}\right)}{\|D_{es}\left(Z_{t}^{es},Z_{s}^{es}\right){\|}_{2}^{2}\cdot \|D_{hs}^{c}\left(Z_{t}^{hs},Z_{s}^{hs}\right){\|}_{2}^{2}}$$

where $D_{es}$ denotes the distance metric in Euclidean space, defined as $D_{es}\left(X,Y\right)=\|X-Y{\|}_{2}^{2}$ , and $D_{hs}^{c}$ represents the distance metric in hyperbolic space, defined as:

$$D_{hs}^{c}\left(X,Y\right)=\frac{1}{\left|c\right|}\cosh^{-1}\left(1-\frac{2c\|X-Y{\|}_{2}^{2}}{\left(1+c\|X{\|}_{2}^{2}\right)\left(1+c\|Y{\|}_{2}^{2}\right)}\right)$$

This consistency loss function ensures the consistency of similar features between Euclidean and hyperbolic spaces by comparing the distances, thereby improving the fusion of multimodal features.

Secondly, in addition to maintaining consistency, it is necessary to enhance the complementarity between different modalities. In this model design, a complementarity loss function $L_{\text{compl}}$ ensures that the label predictions in Euclidean and hyperbolic spaces exhibit complementary relationships, further optimizing the feature fusion effect. The complementarity loss function is defined as follows:

$$\left\{\begin{array}{c} L_{\text{ce}}=D_{\text{ce}}\left({\hat{Y}}^{es},Y^{es}\right)\;\\ L_{\text{compl}}=D_{hs}^{c}\left({\hat{Y}}^{hs},Y^{hs}\right)\; \end{array}\right.$$

where ${\hat{Y}}^{es}$ and ${\hat{Y}}^{hs}$ are the predicted labels in Euclidean and hyperbolic spaces, respectively, and $Y^{es}$ and $Y^{hs}$ are the target labels in Euclidean and hyperbolic spaces. $D_{\text{ce}}$ and $D_{hs}^{c}$ are the distance metric functions in Euclidean and hyperbolic spaces, respectively.

Finally, this paper combines the consistency loss and complementarity loss into a multi-objective optimization problem, with the total loss function $L_{\text{total}}$ represented as follows:

$$L_{\text{total}}=W_{\text{ce}}L_{\text{ce}}+W_{\text{consis}}L_{\text{consis}}+W_{\text{compl}}L_{\text{compl}}$$

where $W_{\text{ce}}$ , $W_{\text{consis}}$ , and $W_{\text{compl}}$ are weight coefficients for each loss term, balancing the losses of consistency, complementarity, and label prediction. This total loss function jointly optimizes features in Euclidean and hyperbolic spaces, allowing the model to better capture the complementarity and consistency of multimodal features, ultimately enhancing diagnostic accuracy and adaptability across diverse diagnostic scenarios.

## 4 Experiments

### 4.1 Experimental setup

#### 4.1.1 Dataset

This paper organizes and utilizes three tongue image datasets from the open-source data platform Roboflow, collecting a total of 4,815 tongue images. Among them, 3,370 images are used as the training set, 722 as the test set, and another 722 as the validation set. The dataset includes a rich variety of features such as tongue coating, tongue body, tongue shape, and tongue edges, characterized by diverse colors, shapes, and thicknesses, providing diversified tongue image information for the model.

This study employs a three-expert annotation mechanism to ensure high-quality and consistent data labeling. First, three experts with extensive clinical experience in TCM were selected, with at least one holding a senior professional title (Associate Chief Physician or above). These experts underwent annotation training, where unified standards were established, and a detailed annotation guideline was developed to ensure a consistent understanding of tongue image features, such as tongue size, thickness, teeth marks, and cracks. During the annotation process, the three experts independently labeled the same tongue image, assigning multi-label information for disease nature and disease location. A consistency check was then conducted—if all three annotations were identical, the data was retained; if discrepancies existed, the data was discarded to prevent noise from affecting model performance. Additionally, to ensure data quality, inter-expert agreement (Cohen’s Kappa) was calculated as a quality evaluation metric, and 10% of the final annotated samples were randomly selected for review, further ensuring the stability and reliability of the data annotation process.

The dataset contains multiple category labels, specifically including:

• Pathological conditions: cold, Qi deficiency, Qi stagnation, heat, dampness, phlegm, blood deficiency, blood stasis, Yang deficiency, and Yin deficiency. To improve annotation accuracy, multiple experts annotated the data. In cases of inconsistent annotations, re-annotation was conducted to ensure only consistent results were retained.

• Pathological locations: intestine, lung, liver, spleen, kidney, stomach, heart, others, and healthy.

**FIGURE 4:**
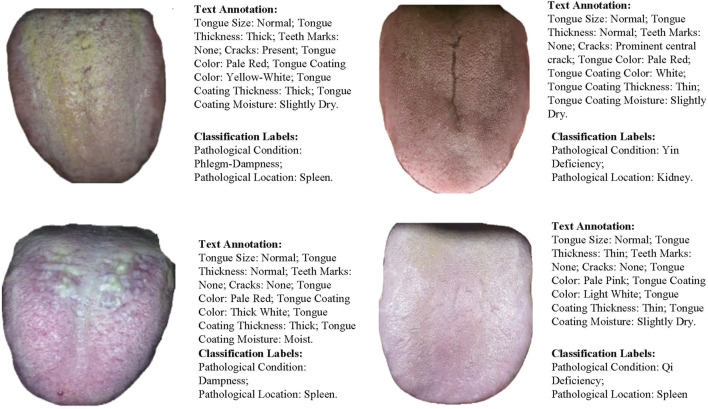
Dataset Sample Display. Including image-text pairs and multi-class label information.

Through this approach, the dataset in this paper encompasses multidimensional features and clinical pathological information of tongue images, providing strong data support for training and testing multimodal diagnostic models. The dataset sample display is shown in Figure 4.

#### 4.1.2 Experimental environment

The experimental environment in this study includes high-performance hardware configurations and software frameworks to ensure the efficiency and stability of model training and testing. The hardware setup is equipped with a multi-core CPU, high-capacity GPU memory, and sufficient RAM, providing support for large-scale data processing and model computation. The software environment utilizes the Ubuntu operating system, combined with Python 3.9 and the PyTorch deep learning framework, with GPU acceleration enabled through CUDA. Additionally, the OpenCV library is integrated for image processing tasks. The specific configurations are shown in Table 1.

**TABLE 1:** Experimental hardware and software Configuration.

Configuration	Name	Specific information
Hardware Environment	CPU	Intel(R) Xeon(R) Gold 6129 CPU @ 2.30 GHz × 32
	GPU	NVIDIA Tesla V100-PCIE × 10
	VRAM	160 GB
	Memory	187 GB
Software Environment	Operating System	Ubuntu
	Python Version	3.9.18
	PyTorch Version	1.13.0
	CUDA Version	11.3
	OpenCV Version	4.6.0

### 4.2 Experimental details

#### 4.2.1 Parameter settings

Table 2 shows the hyperparameter settings used in our experiments to ensure model stability and optimization effectiveness during the training process. These settings include the learning rate, batch size, number of epochs, and optimizer type, which help control the model’s convergence speed and generalization ability.

**TABLE 2:** Hyperparameter settings.

Hyperparameter	Value
Learning Rate	0.001
Batch Size	16
Epochs	300
Optimizer	Adam
Learning Rate Scheduler	Step Decay
Weight Initialization	He Initialization
Dropout	0.5

#### 4.2.2 Evaluation metrics

The evaluation metrics in this paper include Accuracy (Acc), Precision (P), Recall (R), F1 Score (F1), Mean Average Precision (mAP), and Area Under the Curve (AUC). These metrics are used to comprehensively evaluate the classification performance of the model. The formulas are as follows:

$$\begin{array}{cc} \text{Acc} & =\frac{TP+TN}{TP+TN+FP+FN},\\ P & =\frac{TP}{TP+FP},\\ R & =\frac{TP}{TP+FN},\\ F1 & =\frac{2\times P\times R}{P+R},\\ \text{mAP} & =\frac{1}{N}\overset{N}{\underset{i=1}{\sum }}{\text{AP}}_{i},\\ \text{AUC} & =\int_{0}^{1}\text{TPR}\left(t\right)\;d\left(\text{FPR}\left(t\right)\right) \end{array}$$

where TP is the true positive count, TN is the true negative count, FP is the false positive count, FN is the false negative count, N is the total number of classes, ${\text{AP}}_{i}$ is the average precision for class i , TPR(t) is the true positive rate, and FPR(t) is the false positive rate.

### 4.3 Results

As shown in Table 3, TongueNet significantly outperforms existing baseline models, including FocalNet, WaveMLP, ViP, CycleMLP, and LeViT, in both the pathology (Pathology) and location (Location) tasks. Compared to these baseline models, TongueNet demonstrates substantial improvements across multiple evaluation metrics, further validating the effectiveness of the proposed method in multimodal feature fusion and diagnostic tasks.

**TABLE 3:** Performance comparison of TongueNet with other models.

Diagnostic task	Model	Parameters (M)	Evaluation metrics					
			Acc (%)	P (%)	R (%)	F1 (%)	mAP (%)	AUC
Pathology	FocalNet Cao et al. (2019)	31.08	88.44	**68.05**	25.79	36.63	42.16	0.75
	WaveMLP Tang et al. (2022b)	35.42	84.95	44.72	29.92	35.70	35.00	4.18
	ViP Chen et al. (2020)	25.87	82.96	38.93	31.67	34.89	30.74	0.80
	CycleMLP	27.80	87.34	56.37	**31.67**	**40.25**	38.60	0.82
	LeViT Graham et al. (2021)	32.01	88.48	65.45	29.13	39.75	42.84	0.81
	**TongueNet**	32.01	**89.12**	65.82	**38.19**	**48.07**	**46.44**	**0.83**
Location	FocalNet Cao et al. (2019)	31.08	84.53	54.12	35.32	42.58	45.13	0.79
	WaveMLP Tang et al. (2022b)	35.42	83.18	47.86	28.11	35.16	39.29	4.21
	ViP Chen et al. (2020)	25.87	83.86	51.51	36.43	42.57	42.15	0.78
	CycleMLP	27.80	85.24	57.06	**37.17**	**44.85**	44.78	0.81
	LeViT Graham et al. (2021)	32.01	85.55	58.77	35.69	44.18	45.17	0.74
	**TongueNet**	32.01	**86.47**	**62.44**	**39.57**	**48.25**	**47.61**	**0.81**

The bold font represents the optimal result.

In the pathology classification task, TongueNet achieved an accuracy of 89.12%, outperforming FocalNet’s 88.44% and CycleMLP’s 87.34%, showcasing its superior classification capability. Notably, in the critical F1 score metric, TongueNet achieved 48.07%, significantly higher than CycleMLP’s 40.25% and LeViT’s 39.75%, indicating TongueNet’s advantage in balancing precision and recall. Additionally, TongueNet attained 46.44% in mean average precision (mAP) and 0.83 in AUC, both of which are superior to all baseline models. These improvements demonstrate that TongueNet effectively captures subtle pathological features, significantly enhancing the accuracy of pathology classification.

In the location classification task, TongueNet also exhibited exceptional performance, achieving an accuracy of 86.47%, with a clear improvement over CycleMLP and LeViT. In terms of precision, TongueNet achieved 62.44%, significantly higher than FocalNet’s 54.12% and ViP, highlighting its high accuracy in identifying location-specific features. In the F1 score, TongueNet obtained 48.25%, with notable improvements over other models such as CycleMLP and LeViT, further confirming its adaptability and robustness in handling complex feature patterns. TongueNet also achieved 47.61% in mAP and 0.81 in AUC, both outperforming baseline models, demonstrating its stronger ability to understand and differentiate features in location diagnosis tasks.

### 4.4 Ablation study

As shown in Table 4, we evaluated the contribution of the consistency and complementarity modules to the performance of the TongueNet model in both pathology and location diagnosis tasks. The experimental results demonstrate that the inclusion of these modules significantly enhances the model’s performance, further highlighting the advantages of TongueNet in multimodal diagnosis.

**TABLE 4:** TongueNet ablation study on consistency and complementarity.

Diagnostic task	Method	Consistency		Performance metrics					
		Consistency	Complementarity	Acc (%)	P (%)	R (%)	F (%)	mAP (%)	AUC
Pathology	(a)	-	-	84.91	61.02	25.64	36.27	39.71	0.74
	(b)	✓	-	85.45	62.01	28.97	37.47	40.35	0.70
	(c)	-	✓	84.32	58.17	33.28	42.19	42.63	0.71
	(d)	✓	✓	**89.12**	**65.82**	**38.19**	**48.07**	**46.44**	**0.83**
Location	(a)	-	-	82.02	55.24	32.16	40.65	41.64	0.71
	(b)	✓	-	82.34	56.42	33.67	43.18	41.11	0.73
	(c)	-	✓	82.20	55.46	35.67	43.42	41.76	0.75
	(d)	✓	✓	**86.47**	**62.44**	**39.57**	**48.25**	**47.61**	**0.81**

The bold font represents the optimal result.

In the pathology classification task, when the consistency and complementarity modules were not used [configuration (a)], the model achieved an accuracy of 84.91%, an F1 score of 36.27%, and an AUC of 0.74. When the consistency module was introduced alone [configuration (b)], the model’s accuracy improved to 85.45%, with the F1 score increasing by 1.2 percentage points to 37.47%, although AUC slightly decreased to 0.70. This indicates that the consistency module improves some key performance metrics, but the improvement is limited when used alone. When only the complementarity module was added [configuration (c)], the F1 score significantly increased to 42.19%, a 5.92 percentage point improvement over the baseline model (a), and the mAP also improved from 39.71% to 42.63%. In the complete model with both consistency and complementarity modules [configuration (d)], TongueNet achieved an accuracy of 89.12%, the F1 score further increased to 48.07%, and AUC reached 0.83. These results show significant improvements across all metrics compared to the baseline model (a), validating the importance of both modules in capturing multimodal feature complementarity and consistency.

In the location classification task, a similar trend was observed. The baseline model (a), without the consistency and complementarity modules, achieved an accuracy of 82.02%, an F1 score of 40.65%, and an AUC of 0.71. After introducing the consistency module [configuration (b)], the accuracy increased to 82.34%, with slight improvements in the F1 score and AUC, reaching 43.18% and 0.73, respectively. When only the complementarity module was used [configuration (c)], the F1 score reached 43.42%, and AUC increased to 0.75. Finally, in the complete model with both modules [configuration (d)], TongueNet’s accuracy improved to 86.47%, the F1 score increased to 48.25%, and AUC reached 0.81, with significant improvements across all metrics compared to the baseline model.

### 4.5 Analysis of different data proportions

Considering that the effect of consistency and complementarity constraints may be influenced by the dataset size, this section conducts experiments by sampling 25%, 50%, and 100% of the original data to explore the robustness of these spatial constraints with smaller datasets.

As shown in Table 5, when only 25% of the original data is used, the TongueNet model with spatial constraints still outperforms the model without spatial constraints. Specifically, the model’s accuracy improves from 83.19% to 84.05%, and the AUC increases from 0.58 to 0.65. This indicates that even with limited data, spatial constraints still have a positive impact on model performance. With 50% of the data, adding spatial constraints further improves the model’s accuracy to 86.22%, the F1 score rises from 29.48% to 34.45%, and the AUC increases from 0.63 to 0.78. This further validates the effectiveness of spatial constraints with smaller datasets, allowing the model to better capture potential relationships between multimodal features. With the full dataset (100%), the model with spatial constraints achieves the best performance, with an accuracy of 89.12% and an AUC of 0.83, significantly outperforming the configuration without spatial constraints. These results demonstrate that spatial constraints enhance model performance across different data scales, particularly contributing to model robustness and classification accuracy when data is limited.

**TABLE 5:** Effect of dataset size on representational spatial constraints in pathology diagnosis task.

Diagnostic task	Percentage (%)	Spatial constraint	Performance metrics					
			Acc(%)	P (%)	R (%)	F (%)	mAP (%)	AUC
Pathology	25%	No	83.19	36.32	22.64	27.68	32.20	0.58
		Yes	84.05	41.36	25.06	30.97	34.49	0.65
	50%	No	84.71	42.56	22.91	29.48	34.98	0.63
		Yes	86.22	51.23	26.40	34.45	36.77	0.78
	100%	No	84.91	61.02	25.64	36.27	39.71	0.74
		Yes	**89.12**	**65.82**	**38.19**	**48.07**	**46.44**	**0.83**

The bold font represents the optimal result.

A similar trend is observed in the location diagnosis task (as shown in Table 6). With 25% of the data, the model with spatial constraints shows improvements across all metrics compared to the model without spatial constraints, especially with the AUC increasing from 0.61 to 0.65, and a noticeable improvement in the F1 score. When the data increases to 50%, the model with spatial constraints shows a more significant improvement in both accuracy and F1 score, with accuracy reaching 80.35%, compared to 79.30% without spatial constraints. The AUC also shows a significant increase, rising from 0.71 to 0.75. With the full dataset (100%), the introduction of spatial constraints allows the model to achieve optimal performance, with accuracy rising to 86.47% and AUC to 0.81. Overall, spatial constraints contribute significantly to the model’s classification performance across different data scales, especially when data is scarce, improving the model’s robustness and generalization ability. These experimental results further demonstrate the potential of TongueNet in multimodal traditional Chinese medicine diagnosis, as it can effectively capture the relationship between tongue images and textual features, even with limited data, thus improving diagnostic accuracy.

**TABLE 6:** Effect of dataset size on representational spatial constraints in location diagnosis task.

Diagnostic task	Percentage (%)	Multi-display constraint	Performance metrics					
			Acc(%)	P (%)	R (%)	F (%)	mAP (%)	AUC
Location	25%	No	75.44	31.07	19.64	24.07	34.04	0.61
		Yes	77.64	37.50	19.29	25.47	36.12	0.65
	50%	No	79.30	46.15	21.05	28.92	39.73	0.71
		Yes	80.35	51.55	29.24	37.31	43.18	0.75
	100%	No	82.02	55.24	32.16	40.65	41.64	0.71
		Yes	**86.47**	**62.44**	**39.57**	**48.25**	**47.61**	**0.81**

The bold font represents the optimal result.

### 4.6 Comparison of multimodal and unimodal

To further validate the effectiveness of multimodal fusion in TongueNet for pathology and location diagnosis tasks, this paper compares the performance of the multimodal (image and text) model with the unimodal (image-only) model.

As shown in Table 7, in the pathology diagnosis task, the multimodal model outperforms the unimodal model across all metrics. Specifically, the accuracy of the unimodal model is 81.87%, while the multimodal model reaches 89.12%, improving by 7.25 percentage points. At the same time, the precision and F1 score of the multimodal model improved by approximately 3.4% and 1.94%, respectively. In terms of mean average precision (mAP) and AUC, the multimodal model also showed significant advantages, reaching 46.44% and 0.83, which is an improvement of 18.15% and 0.05 over the unimodal model. This indicates that multimodal information fusion can effectively enhance the model’s ability to capture pathology features, leading to more accurate diagnostic results.

**TABLE 7:** Comparison of TongueNet’s pathology diagnosis performance between multimodal and unimodal models.

Training mode	Modality	Acc(%)	P (%)	R (%)	F (%)	mAP (%)	AUC
Unimodal	Image	81.87	62.42	39.15	46.13	28.29	0.78
Multimodal	(Image & Text)	89.12	65.82	38.19	48.07	46.44	0.83

As shown in Table 8, in the location diagnosis task, the multimodal model also demonstrates better performance. The accuracy of the unimodal model is 84.63%, while the multimodal model improves to 86.47%. In terms of the F1 score, the multimodal model reaches 48.25%, improving by 12.49 percentage points, which indicates that multimodal fusion significantly improves the robustness and accuracy of the model for location diagnosis. Additionally, the mAP improves from 26.24% in the unimodal model to 47.61%, and the AUC increases from 0.75 to 0.81, further proving the advantage of multimodal features in capturing location-specific characteristics.

**TABLE 8:** Comparison of TongueNet’s location diagnosis performance between multimodal and unimodal models.

Training mode	Modality	Acc(%)	P (%)	R (%)	F (%)	mAP (%)	AUC
Unimodal	Image	84.63	60.32	38.47	35.76	26.24	0.75
Multimodal	(Image & Text)	86.47	62.44	39.57	48.25	47.61	0.81

### 4.7 Discussion

The proposed TongueNet employs a multimodal deep learning approach to integrate tongue image analysis and textual features, significantly enhancing the automation of Traditional Chinese Medicine (TCM) tongue diagnosis. In disease nature and lesion location classification tasks, the model outperforms existing traditional methods across multiple key evaluation metrics, such as accuracy and AUC. The findings of TongueNet have broad implications for clinical applications and the development of AI in healthcare. Tongue analysis is a crucial component of TCM diagnosis, traditionally relying on practitioners’ subjective experience, which often leads to inconsistencies among different physicians. This study addresses this issue by standardizing and quantifying tongue features through a data-driven approach, reducing human-induced errors and enhancing diagnostic consistency. Furthermore, TongueNet surpasses the limitations of traditional single-modal tongue diagnosis methods by achieving joint learning of image and text features, enabling more comprehensive and accurate disease nature and lesion location diagnosis. Compared to rule-based approaches, TongueNet exhibits greater adaptability and self-learning capability, continuously improving its generalization performance with new data, making it suitable for tongue diagnosis tasks across different regions and patient groups. This method can assist doctors in hospitals and clinics in making rapid diagnoses and can also be applied to telemedicine systems. It is particularly beneficial for primary healthcare institutions and remote areas, enabling efficient, low-cost intelligent tongue diagnosis, thereby improving access to medical resources.

Although TongueNet has made significant progress in the automation of TCM tongue diagnosis, this study still has certain limitations. One of the primary bottlenecks affecting the model’s generalization ability is the insufficient scale and diversity of the dataset. The dataset used in this study consists of 4,815 tongue images, which is relatively small compared to other mainstream medical imaging datasets, such as CheXpert and ImageNet. This data limitation may lead to performance instability in different populations or clinical settings, especially when dealing with individuals of different ethnicities, ages, genders, and lifestyles. For instance, research indicates that dietary habits, regional climate, and genetic factors influence tongue characteristics. However, the current dataset does not encompass a sufficiently diverse range of individuals, which may restrict the model’s applicability to certain populations. Therefore, future research should focus on expanding the dataset size and developing a multimodal tongue image database that includes diverse geographic regions and populations to enhance the model’s adaptability and robustness.

Furthermore, the complex relationship between disease nature and lesion location may not have been fully explored. For example, certain disease natures (e.g., “Spleen Qi Deficiency”) are often highly correlated with specific lesion locations (e.g., “pale and swollen tongue”), but the current model does not explicitly capture these pathological associations and instead treats them as two independent classification tasks. Future research can enhance the model’s clinical applicability by incorporating additional annotation dimensions, such as microscopic tongue features (thickness of tongue coating, cracks, moisture levels), syndrome combinations (changes in Qi, blood, and body fluids), and individual health conditions (dietary habits, lifestyle factors). Additionally, introducing Graph Neural Networks (GNNs) or relation inference models can help explore the structured relationships between disease nature, lesion location, and syndrome types, enhancing the model’s understanding of complex pathological patterns and improving diagnostic reasoning and medical interpretability.

The application of artificial intelligence in medical diagnosis still faces ethical and interpretability challenges. Although TongueNet has demonstrated excellent performance in experiments, its decision-making process remains a “black box,” lacking interpretability. Both doctors and patients may find it difficult to understand the basis of AI diagnoses, which could affect the acceptance and trustworthiness of AI-assisted diagnosis in real-world medical practice. Moreover, in clinical practice, the issue of responsibility attribution for AI diagnosis remains an unresolved ethical concern. Currently, most medical AI systems operate as Clinical Decision Support (CDS) systems, where the final diagnostic decision is made by the physician. However, if an AI misdiagnosis occurs, there is no clear consensus on whether the physician should bear full responsibility for the error.

Future research can be optimized and expanded in multiple directions. First, expanding the dataset size and diversity is crucial by collecting tongue images from individuals of different genders, ages, regions, and dietary habits, thereby constructing a more representative multimodal medical imaging database. Second, in terms of model optimization, integrating Transformer architectures can enhance cross-modal information interaction, while incorporating self-supervised learning (SSL) methods can reduce dependence on large-scale manually annotated data. Additionally, to improve the credibility of AI diagnosis, future studies should focus on explainable AI (XAI) methods, such as Grad-CAM, LIME, and SHAP, enabling physicians to intuitively understand AI’s diagnostic logic, thereby increasing clinicians’ trust in AI-assisted diagnosis. Finally, TongueNet can be further integrated with telemedicine systems and smart health devices to enable real-time tongue diagnosis analysis and facilitate the development of mobile AI-based tongue diagnosis systems, promoting the clinical application of intelligent TCM diagnosis.

## 5 Conclusion

This paper presents a multimodal deep learning model called TongueNet, which combines tongue image and text information to achieve high-precision multi-label classification for pathology and location in TCM diagnosis. TongueNet utilizes a HAN and a feature space projection module to efficiently extract and integrate multimodal features. The model applies consistency and complementarity constraints to optimize the fusion of tongue and text features. Additionally, the EMA attention mechanism is introduced to effectively allocate weights across multimodal features, enhancing the diversity and accuracy of feature representation. TongueNet also replaces the traditional MLP with a KAN for output optimization. KAN’s multi-level nonlinear function learning strengthens the model’s ability to represent complex features, further improving classification performance. Experimental results show that TongueNet outperforms existing models in both pathology and location diagnosis tasks, validating its potential application in multimodal TCM diagnosis.

## Data Availability

The original contributions presented in the study are included in the article/supplementary material, further inquiries can be directed to the corresponding author.
